# The Risk of Major Adverse Cardiovascular Events in Ankylosing Spondylitis Patients With a History of Acute Anterior Uveitis: A Nationwide, Population Based Cohort Study

**DOI:** 10.3389/fmed.2022.884800

**Published:** 2022-07-07

**Authors:** Yi-Chiao Bai, Chin-Hsiu Liu, Pui-Ying Leong, Kuo-Lung Lai, Hsin-Hua Chen, James Cheng-Chung Wei

**Affiliations:** ^1^Institute of Medicine, Chung Shan Medical University, Taichung, Taiwan; ^2^Department of Optometry, Shu-Zen Junior College of Medicine and Management, Kaohsiung, Taiwan; ^3^Division of Allergy, Immunology and Rheumatology, Department of Internal Medicine, Chung Shan Medical University Hospital, Taichung, Taiwan; ^4^Rheumatology and Immunology Center, China Medical University Hospital, Taichung, Taiwan; ^5^Division of Allergy, Immunology and Rheumatology, Department of Internal Medicine, Taichung Veterans General Hospital, Taichung, Taiwan; ^6^Department of Medical Research, Taichung Veterans General Hospital, Taichung, Taiwan; ^7^Department of Industrial Engineering and Enterprise Information, Tunghai University, Taichung, Taiwan; ^8^School of Medicine, National Yang-Ming University, Taipei, Taiwan; ^9^Institute of Biomedical Science and Rong Hsing Research Centre for Translational Medicine, Chung Hsing University, Taichung, Taiwan; ^10^Graduate Institute of Integrated Medicine, China Medical University, Taichung, Taiwan

**Keywords:** ankylosing spondylitis (AS), acute anterior uveitis (AAU), cohort study, major adverse cardiovascular events (MACE), autoimmune disease

## Abstract

**Background:**

To investigate the association between a history of acute anterior uveitis (AAU) and the risk of major adverse cardiovascular events (MACE) among patients with ankylosing spondylitis (AS).

**Methods:**

We identified 38,691 newly diagnosed AS patients between 2003 and 2013 from the Taiwan National Health Insurance Research Database. The exposure group was defined as people with uveitis diagnosis by ophthalmologist before AS diagnosis date. The incidence of MACE in patients with AS according to the International Classification of Diseases, Ninth Revision. We randomly selected a comparison group without a history of AAU at a 1:4 ratio matched by age, sex, and index year in relation to the risk of developing MACE. We used cox proportional hazard regression model to compare the risk of MACE between groups, shown as adjusted hazard ratios (aHRs) with 95% confidence intervals (CI). Further subgroup analysis and sensitivity tests were also performed.

**Results:**

There were 3,544 patients in the AAU group and 14,176 patients in the non-AAU group. The aHR of MACE for the AAU group was 0.79 (95% CI = 0.57–1.10) at a 1:4 ratio for age, sex and index year. Sensitivity analyses using various adjustment variables showed consistent results. Cox proportional hazard regression model demonstrated that use of non-steroidal anti-inflammatory drugs (NSAIDs) was associated with an increased risk of MACE in this cohort (HR = 3.44; 95% CI = 2.25–5.25).

**Conclusion:**

This cohort study showed that subjects with AAU was not associated with the risk of MACE among AS patients, compared to non-AAU controls.

## Introduction

Ankylosing spondylitis (AS) is a progressive rheumatic disease that affects spine and peripheral joints, leading to disabilities (1, 2). The prevalence of AS varies from 0.6% to 1.4% in Western populations, and the prevalence of AS in Taiwan is 96.9 cases per 100,000 people (3). The increased risk of cardiovascular disease among AS patients has been well-established, and patients diagnosed with AS have been found to be at higher risk of developing cardiovascular event (4, 5). Among patients with AS, cardiovascular risk is associated with hypertension, high body mass index, smoking, and low physical activity (6). Toll-like receptor (TLR) 2 and TLR4 are microbial sensors and promotors for atherosclerosis, leading to atheroma development and progression, and the development of cardiovascular disease (7).

Acute anterior uveitis (AAU) is the most common form of uveitis, which is characterized by one of the extra-articular manifestations of AS. It is estimated that the incidence of uveitis is 17 and 52.4 cases per 100,000 people, about 5.5% of newly-diagnosed AS patients had AAU before the diagnosis of AS (8). The factors of uveitis may be caused by the infectious agent or related to immune-mediated diseases. Chang JH et al. revealed a significant reduction in TLR2 expression levels on monocytes and neutrophils of patients with AAU (9). However, the association of major cardiovascular event (MACE) with a history of AAU in AS patients has not yet been clarified.

The National Health Insurance Research (NHIRD) had facilitated a population-based longitudinal epidemiological study in Taiwan. Therefore, the present study aimed to assess the correlation between AAU and the risk of MACE in incident AS patients.

## Methods

### Data Source

The data was drawn from the National Health Insurance Research Database (NHIRD) of Taiwan, which has been expanded to cover the medical needs of citizens of the Taiwanese population, i.e., ~99% of the population of Taiwan. These data were linked to the Longitudinal Health Insurance Database (LHID) 2000 in Taiwan. This population-based retrospective cohort study used data from January 1, 2003, to December 31, 2013. NHIRD is one of the largest administrative medical databases in the world and has been widely used in academic research. It is a powerful resource for observing chronic diseases and disease diagnosis. Each subject is encoded with an encrypted identifier, which can be used to link future patient data, medications prescribed, demographic data, socioeconomic status, and location of patients (10). The data used from the NHIRD was anonymized before analyses, so no informed consent was required.

### Definition of AS

We considered individuals who had at least three outpatient visits with a diagnosis of AS (International Classification of Diseases, Ninth Revision, Clinical Modification [ICD-9-CM] as patients with AS. We identified 38,691 newly-diagnosed AS patients from 2003 to 2013. The index date was AS patients who never had a diagnosis of uveitis. Follow-up started on the index date and ended at either MACE occurrence, withdrawal from the NHI due to any cause such as death or leaving, or the end of the dataset (31 December 2013).

### Definition of a History of AAU

**Figure 1:**
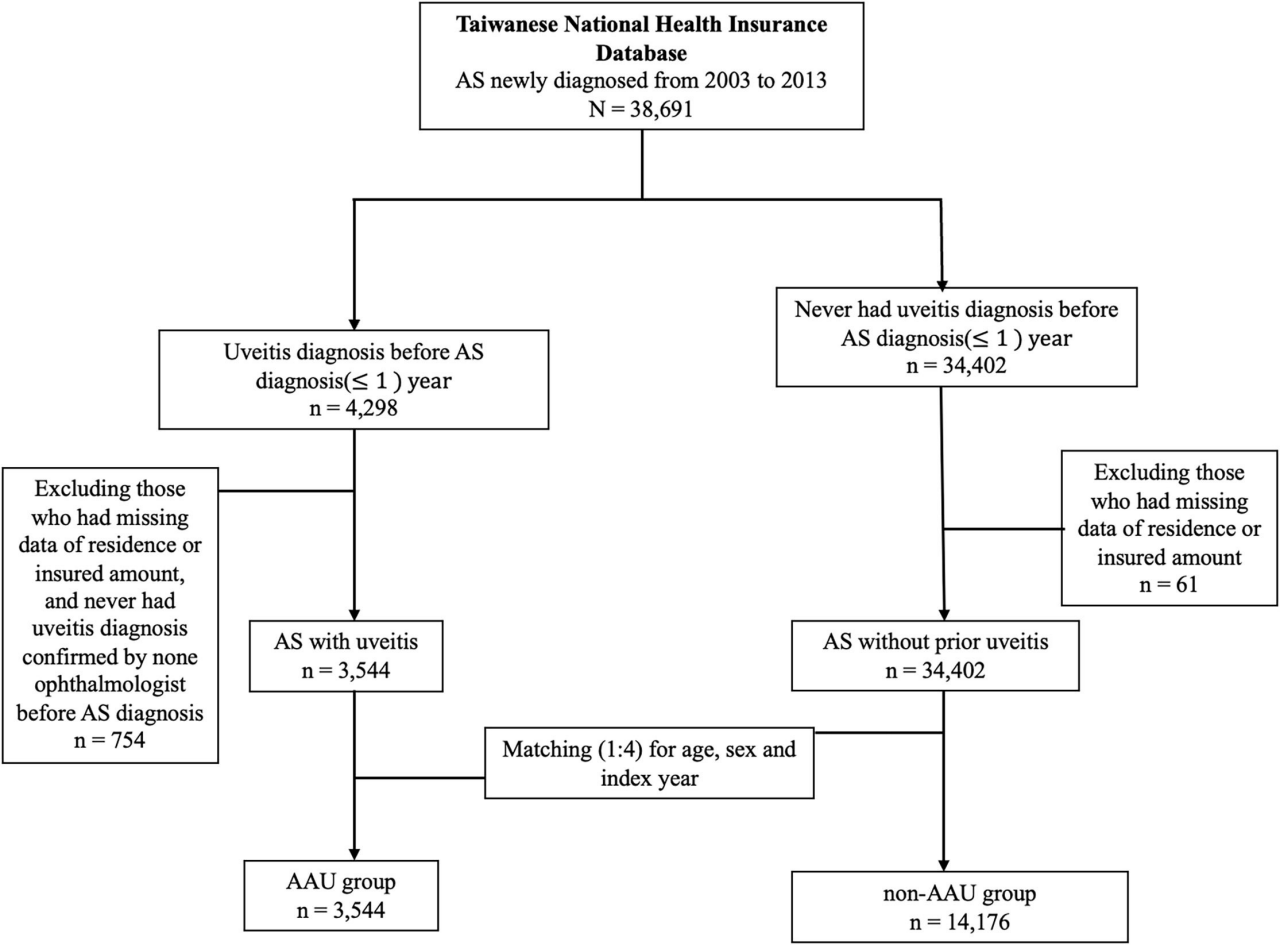
Flow chart of inclusion criteria.

We defined a history of AAU as having at least one inpatient or ambulatory visits with a diagnosis of AAU (ICD-9-CM codes 364–364.02, 364.04–364.05, 364.3) made by a qualified ophthalmologist before the first date of uveitis diagnosis. We therefore identified 3,544 AS patients with a history of AAU (AAU group) from all newly-diagnosed AS patients after excluding 4 patients with missing data of residence or insured amount. We matched the AAU group with AS patients without a history of AAU (non-AAU group) at a 1:4 ratio for age, sex and index year. Finally, we included 3,544 AS patients in the AAU group and 14,176 AS patients in the non-AAU group (Figure 1).

### Outcome

The outcome of the study was the time from the first data of AS diagnosis to the development of MACE. We defined MACE as having been hospitalized for at least 3 days (unless patients died) with a diagnosis of myocardial infarction (MI) (ICD-9-CM codes 410.x, except 410.x2) with receiving a procedure of coronary artery by pass graft (CABG) (ICD-9-CM code 36.1, 36.2) or percutaneous coronary intervention (PCI)/percutaneous transluminal coronary angioplasty (PTCA)/stent (ICD-9-CM code 00.66, 36.03, 36.06, 36.07, 36.09), or with a hospital discharge diagnosis of ischemic stroke (ICD-9-CM code 433–436, except 433.x0 and 434.x0). The diagnosis of MACE using aforementioned criteria been verified in previous database research (11, 12). There were 43 cases of MACE in the AAU-group, and there were 226 case of MACE in the Non-AAU group among AS patients (**Table 2**).

### Potential Confounders

Potential confounders included age, sex, urbanization level of residence and comorbidities at baseline, and average doses of medications for AS treatment during the follow-up period. Comorbidity was identified up to 1 year before the index date, including heart failure *(*ICD-9-CM code 428), hypertension (ICD-9-CM codes 401–405), diabetes mellitus (ICD-9-CM code 250), hyperlipidemia (ICD-9-CM code 272), ischemic heart disease (ICD-9-CM codes 410–414), ischemic stroke (ICD-9-CM code 433–436, except 433.x0 and 434.x0), chronic obstructive pulmonary disease (COPD), asthma, interstitial lung disease) (ICD-9-CM code COPD: 49X, 500–505, 506.4; asthma: 493; ILD: 515, 516.3, 517, 516.8, 516.9), chronic kidney disease (ICD-9-CM code 582, 583.0-583.7, 585, 586, 588), chronic liver disease (ICD-9-CM code 5712, 5714, 5715, 5716, 4560, 4561, 4562, 572.2–572.8), hyperthyroidism (ICD-9-CM code 242), inflammatory bowel disease (ICD-9-CM code 555–556), psoriasis (ICD-9-CM code 696.1), and anti-phospholipid syndrome (ICD-9-CM code 289.8).

AS treatment was identified during the follow-up period. AS patients often received NSAID and/or traditional disease-relieving anti-rheumatic drug (DMARD) treatment. To avoid immortal time bias, we used average cumulative defined daily dose (DDD) of NSAIDs during the follow-up period [i.e., cumulative DDD (cDDD)/follow-up duration (days)] to control the influence of the confounding effect of NSAIDs. Likewise, according to the definition of the World Health Organization (WHO), we further explored the classification of these data as a hypothetical mean maintenance dose, including methotrexate (MTX) per 2.5 mg/week, sulfasalazine per 500 mg/day, corticosteroid (prednisolone equivalent dose, mg/day), etanercept per 200 mg/4 weeks, adalimumab per 80 mg/4 weeks, and golimumab per 50 mg/4 weeks.

### Statistical Analysis

Demographic characteristic data, including the distributions of categorical age, sex, urbanization level, income level, length of previous hospital stays, medical comorbidities, and co-medications between the AAU study group and non-AAU comparison group were analyzed by chi-square (v2) tests. We then used absolute standardized difference (ASD) to define patient characteristics as well-balanced (ASD < 0.1), that is, a standardized mean difference of <0.1 between AAU and non-AAU groups, and there was no significant difference between the two groups for most clinical manifestations and comorbidities. The incidence density of MACE per 100,000 person years was calculated in both groups. The Kaplan–Meier method was used to describe the cumulative incidence of MACE in the two groups; differences between the two groups were evaluated using the log-rank test. We used Cox proportional hazed regression for estimation of adjusted hazard ratio (aHR) and 95% confidence interval (CI). Four statistical models were fitted to evaluate the effect of AAU on the risk of MACE. The first model (Model 1) only assessed the crude effect of AAU on the risk of incidental MACE. In the second model (Model 2), we used demographic variables, including sex, age, urbanization, and low income at baseline. In the third model (Model 3), we examined the temporal relationship between AAU exposure and the risk of developing MACE for both cohorts, adjusted for demographic variables, length of hospital stays, and comorbidities at baseline. Our four model (Model 4) adjusted for demographic variables, length of hospital stays, comorbidities, and co-medications at baseline (continuous variable). Finally, in the fifth model (Model 5), we adjusted for demographic variables, length of hospital stays, and comorbidities at baseline (categorical variable). We used the SAS statistical software package, version 9.3 (SAS Institute, Cary, NC) for analysis. The level of statistical significance was set at a *p* < 0.05.

## Results

### Demographic Baseline Data of the Enrolled Patients

To demonstrate the impact of baseline characteristics of the participants at follow-up period, we used age, sex, and index date matching to examine the characteristics at baseline. There were 3,544 patients in the AAU group and 14,176 patients in the non-AAU group at a 1:4 ratio by using age and sex and index date for comparison. The results also showed that the small differences in the AAU and non-AAU groups using ASD analysis, as shown in Table 1.

**Table 1:** Baseline characteristics among AAU group and non-AAU group.

	**Before PSM (1:4 age–sex matching)**				**1:1 PSM**			
	**Non-AAU**	**AAU**	***P*-value**	**ASD**	**Non-AAU**	**AAU**	***P*-value**	**ASD**
	***n* = 14,176**	***n* = 3,544**			***n* = 3,540**	***n* = 3,540**		
**Follow-up**	3.9 ± 1.8	3.8 ± 1.9	<0.001		3.9 ± 1.8	3.8 ± 1.9	<0.001	
**Sex**			1	0			1	0
Female	4,948 (34.9)	1,237 (34.9)			1,236 (34.9)	1,236 (34.9)		
Male	9,228 (65.1)	2,307 (65.1)			2,304 (65.1)	2,304 (65.1)		
**Age**	40.9 ± 13.9	40.9 ± 13.9	1	0	40.8 ± 13.8	40.9 ± 13.9	0.705	0.009
<30	3,248 (22.9)	812 (22.9)	1		811 (22.9)	811 (22.9)	1	
30–45	5,464 (38.5)	1,366 (38.5)			1,365 (38.6)	1,365 (38.6)		
45–65	4,704 (33.2)	1,176 (33.2)			1,174 (33.2)	1,174 (33.2)		
≥65	760 (5.4)	190 (5.4)			190 (5.4)	190 (5.4)		
**Urbanization**		<0.001	0.075			0.637	0.015
Urban	4,404 (31.1)	1,186 (33.5)			1,175 (33.2)	1,183 (33.4)		
Suburban	6,986 (49.3)	1,761 (49.7)			1,738 (49.1)	1,760 (49.7)		
Rural	2,786 (19.7)	597 (16.8)			627 (17.7)	597 (16.9)		
**Low income (≤Q2:21,900)**	8,161 (57.6)	1,926 (54.3)	<0.001	0.065	1,936 (54.7)	1,924 (54.4)	0.775	0.007
Length of hospital stays^*^	2.5 ± 15.2	1.9 ± 15.4	0.046	0.074	2.1 ± 14.2	1.9 ± 15.4	0.617	0.012
0 day	11,693 (82.5)	2,993 (84.5)	<0.001		3,031 (85.6)	2,991 (84.5)	0.042	
1–6 days	1,356 (9.6)	346 (9.8)			287 (8.1)	345 (9.7)		
≥7 days	1,127 (8.0)	205 (5.8)			222 (6.3)	204 (5.8)		
**Co-morbidity^†^**							
Heart failure	78 (0.6)	23 (0.6)	0.485	0.013	17 (0.5)	22 (0.6)	0.422	0.019
Hypertension	1,670 (11.8)	444 (12.5)	0.219	0.023	410 (11.6)	442 (12.5)	0.242	0.028
Diabetes mellitus	689 (4.9)	149 (4.2)	0.1	0.032	138 (3.9)	149 (4.2)	0.507	0.016
Hyperlipidemia	1,203 (8.5)	289 (8.2)	0.525	0.012	247 (7)	288 (8.1)	0.065	0.044
Ischemic heart disease	455 (3.2)	100 (2.8)	0.236	0.023	76 (2.1)	100 (2.8)	0.067	0.044
Ischemic stroke	122 (0.9)	24 (0.7)	0.28	0.021	28 (0.8)	24 (0.7)	0.578	0.013
Pulmonary disease	437 (3.1)	101 (2.8)	0.47	0.014	103 (2.9)	101 (2.9)	0.887	0.003
Chronic kidney disease	132 (0.9)	20 (0.6)	0.034	0.043	16 (0.5)	20 (0.6)	0.504	0.016
Chronic liver disease	408 (2.9)	81 (2.3)	0.054	0.037	65 (1.8)	81 (2.3)	0.181	0.032
Hyperthyroidism	83 (0.6)	16 (0.5)	0.338	0.019	15 (0.4)	16 (0.5)	0.857	0.004
IBD	34 (0.2)	3 (0.1)	0.07	0.039	3 (0.1)	3 (0.1)	1	0
Psoriasis	111 (0.8)	24 (0.7)	0.517	0.012	21 (0.6)	24 (0.7)	0.654	0.011
APS	0 (0.0)	2 (0.1)	0.005	NA	0 (0.0)	0 (0.0)	NA	NA
**AS treatment at baseline^‡^**						
NSAID, cDDD/day	0.2 ± 0.2	0.3 ± 0.3	<0.001		0.2 ± 0.2	0.3 ± 0.3	<0.001	
Methotrexate (cdose/2.5mg) week	0.1 ± 0.8	0.1 ± 0.4	0.308		0.1 ± 0.4	0.1 ± 0.4	0.024	
Sulfasalazine (cdose/500mg)/day	0.2 ± 0.5	0.4 ± 0.7	<0.001		0.2 ± 0.5	0.4 ± 0.7	<0.001	
Steroid, mg/day	0.4 ± 7.0	0.6 ± 1.6	0.004		0.4 ± 2.0	0.6 ± 1.6	<0.001	
Etanercept,200mg/4week	0.005 ± 0.1	0.01 ± 0.1	<0.001		0.005 ± 0.05	0.01 ± 0.1	<0.001	
Adalimumab,80mg/4week	0.005 ± 0.1	0.01 ± 0.1	<0.001		0.01 ± 0.1	0.01 ± 0.1	<0.001	
Golimumab, 50mg/4week	0.0004 ± 0.01	0.001 ± 0.03	0.048		0.001 ± 0.01	0.001 ± 0.03	0.146	
NSAID	13,679 (96.5)	3,394 (95.8)	0.039		3,416 (96.5)	3,390 (95.8)	0.109	
Methotrexate	958 (6.8)	321 (9.1)	<0.001		244 (6.9)	321 (9.1)	<0.001	
Sulfasalazine	5,604 (39.5)	2,297 (64.8)	<0.001		1,418 (40.1)	2,294 (64.8)	<0.001	
Steroid	7,823 (55.2)	2,503 (70.6)	<0.001		1,947 (55.0)	2,500 (70.6)	<0.001	
Etanercept	181 (1.3)	85 (2.4)	<0.001		48 (1.4)	85 (2.4)	0.001	
Adalimumab	200 (1.4)	109 (3.1)	<0.001		56 (1.6)	109 (3.1)	<0.001	
Golimumab	30 (0.2)	17 (0.5)	0.006		11 (0.3)	17 (0.5)	0.256	

* *Length of hospital stay was identified within 2 years before index date*.

† *Comorbidity was identified within 1 year before index date*.

‡ *The definition of “AS treatment” refers to the utilization of related medication between the day of first having AS diagnosis and the endpoint (end of follow-up)*.

### Comparison the Incidence of MACE Among AAU Groups and Non-AAU Groups

Table 2 compares the incidence of MACE in the AAU and non-AAU groups. Results showed that the overall crude rate of MACE was 323.48 per 100,000 person-years in the AAU group, and 407.24 per 100,000 person-years in the non-AAU group during follow-up duration. The crude relative risk of the uveitis group among the 13,293 assessed in the follow-up was found to be 0.79 (95% CI = 0.57–1.10). The results showed that patients with uveitis did not increase the risk of MACE among AS patients.

**Table 2:** Incidence of MACE in PSM study group.

	**Before PSM (1:4 age–sex matching)**		**1:1 PSM**	
	**Non-AAU**	**AAU**	**Non-AAU**	**AAU**
*n*	14,176	3,544	3,540	3,540
Follow-up person years	55,495	13,293	13,794	13,277
MACE	226	43	50	43
Incidence rate^*^ (95%CI)	407.24 (407.07–407.41)	323.48 (323.17–323.78)	362.48 (362.16–362.80)	323.86 (323.56–324.17)
Crude relative risk (95%CI)	Ref.	0.79 (0.57–1.10)	Ref.	0.89 (0.59–1.34)
Average Follow-up duration	3.9	3.8	3.9	3.8

* *Incidence rate, per 100,000 person-years*.

### Sensitivity Analysis for Estimating the aHR of MACE

As shown in Table 3, to minimize the potential confounding effects of age, sex and selected comorbidities on the incidence of MACE among AS patients with AAU, we performed cox proportional hazard regression for estimation of aHR of MACE for AAU Model 1 to Model 4. We used age- and sex-matching the population, the results showed that Model 1—AAU exposure alone does not increase the risk of MACE development (aHR = 0.79, 95% CI = 0.57–1.10). Model 2—AAU exposure and demographic variables showed that MACE risk for AAU was 0.81 (95% CI= 0.59–1.13). The Cox proportional hazard regression for estimation of aHR of MACE in Model 3—Model 2 + medical utilization and comorbidities at baseline was 0.82 (95% CI = 0.59–1.14). The data showed Model 4—model 3 + AS treatment was not significantly the risk of MACE for AAU (aHR = 0.82, 95% CI = 0.59–1.14), and data showed Model 5—model 3 + AS treatment indicated that MACE risk for AAU was 0.98 (95% CI = 0.70–1.37). To determine the effect of treatment on AS patients and the incidence of MACE, we again performed the analysis, and found that NSAID was associated with MACE (aHR = 3.44, 95% CI = 5.07–11.12). Furthermore, the treatment responses to sulfasalazine (cdose/500 mg)/day indicated a lower incidence of MACE among patients with AS in the follow-up period in this cohort study (aHR = 0.68, 95% CI = 0.49–0.93). In Table 4, we used the time dependent cox regression for estimation of adjusted HRs on MACE, there is no difference in the risk of MACE at 1:4 age-matched and sex-matched population.

**Table 3:** Cox proportional hazard regressions for 1:4 age-matched and sex-matched population estimation of adjusted HRs on MACE.

	**Model 1: AAU exposure alone**	**Model 2: AAU exposure +demographic variables**	**Model 3: model 2+medical utilization and comorbidities at baseline**	**Model 4: model 3+AS treatment (continuous variable)**	**Model 5: model 3+AS treatment (categorical variable)**
**AAU**	0.79 (0.57–1.10)	0.81 (0.59–1.13)	0.82 (0.59–1.14)	0.82 (0.59–1.14)	0.98 (0.70–1.37)
**Sex- Male**		2.87 (2.11–3.90)	2.59 (1.90–3.52)	2.64 (1.94–3.60)	2.51 (1.84–3.42)
Age					
<30		Reference	Reference	Reference	Reference
30–45		7.60 (2.74–21.10)	6.67 (2.40–18.54)	6.56 (2.36–18.22)	6.49 (2.33–18.05)
45–65		28.95 (10.73–78.10)	16.95 (6.22–46.18)	16.02 (5.87–43.70)	15.76 (5.77–43.00)
≥65		90.39 (32.86–248.67)	29.80 (10.56–84.15)	28.20 (9.97–79.78)	24.85 (8.76–70.52)
**Urbanization**					
Urban		Reference	Reference	Reference	Reference
Suburban		1.05 (0.79–1.40)	1.02 (0.76–1.36)	1.00 (0.75–1.34)	1.05 (0.78–1.40)
Rural		1.07 (0.76–1.50)	1.01 (0.72–1.41)	0.96 (0.69–1.35)	1.08 (0.77–1.51)
**Low income**		1.61 (1.21–2.15)	1.41 (1.05–1.90)	1.36 (1.01–1.83)	1.41 (1.05–1.89)
Length of hospital stays^*^					
0 day			Reference	Reference	Reference
1–6 days			1.66 (1.19–2.32)	1.64 (1.18–2.29)	1.68 (1.21–2.35)
≥7 days			1.84 (1.33–2.54)	1.80 (1.30–2.50)	1.79 (1.29–2.49)
**Co-morbidity^†^**					
Heart failure			1.50 (0.84–2.70)	1.41 (0.78–2.55)	1.46 (0.81–2.66)
Hypertension			1.83 (1.36–2.47)	1.75 (1.30–2.36)	1.83 (1.35–2.47)
Diabetes mellitus			1.70 (1.23–2.34)	1.72 (1.25–2.37)	1.67 (1.21–2.31)
Hyperlipidemia			1.42 (1.04–1.93)	1.44 (1.06–1.95)	1.40 (1.03–1.91)
Ischemic heart disease			1.97 (1.41–2.76)	2.03 (1.45–2.84)	2.04 (1.45–2.87)
Ischemic stroke			1.67 (0.99–2.83)	1.80 (1.06–3.05)	1.66 (0.98–2.81)
Pulmonary disease			1.20 (0.77–1.88)	1.12 (0.72–1.75)	1.25 (0.80–1.96)
Chronic kidney disease			1.65 (0.93–2.94)	1.65 (0.92–2.96)	1.62 (0.91–2.89)
Chronic liver disease			0.73 (0.39–1.38)	0.73 (0.38–1.37)	0.73 (0.39–1.38)
IBD			0.55 (0.07–4.25)	0.21 (0.03–1.78)	0.60 (0.08–4.70)
Psoriasis			0.42 (0.06–3.01)	0.37 (0.05–2.72)	0.48 (0.07–3.50)
**AS treatment during f/u period^‡^**					
NSAID, cDDD/day				3.44 (2.25–5.25)	
Methotrexate (cdose/2.5 mg) week				0.94 (0.67–1.32)	
Sulfasalazine (cdose/500 mg)/day				0.68 (0.49–0.93)	
Steroid, mg/day				1.008 (1.005–1.010)	
**AS treatment during f/u period^‡^**					
NSAID					0.32 (0.20–0.50)
Methotrexate					1.24 (0.70–2.20)
Sulfasalazine					0.63 (0.46–0.86)
Steroid					0.63 (0.49–0.81)

* *Length of hospital stay was identified within 2 years before index date*.

† *Comorbidity was identified within 1 year before index date*.

‡ *AS treatment was identified after diagnosis with AS*.

**Table 4:** Time dependent cox regression 1:4 age-matched and sex-matched population for estimation of adjusted HRs on MACE.

	**Model 1: AAU exposure alone**	**Model 2: AAU exposure +demographic variables**	**Model 3: model 2+medical utilization and comorbidities at baseline**	**Model 4: model 3+AS treatment (continuous variable)**	**Model 5: model 3+AS treatment (categorical variable)**
**AAU**	0.79 (0.57–1.10)	0.81 (0.59–1.13)	0.82 (0.59–1.14)	0.80 (0.57–1.12)	0.85 (0.61–1.18)
**Sex- Male**		2.87 (2.11–3.90)	2.59 (1.90–3.52)	2.62 (1.92–3.57)	2.58 (1.90–3.51)
Age					
<30		Reference	Reference	Reference	Reference
30–45		7.61 (2.74–21.13)	6.67 (2.40–18.54)	6.55 (2.36–18.21)	6.62 (2.38–18.39)
45–65		28.98 (10.73–78.23)	16.95 (6.22–46.18)	16.08 (5.90–43.87)	16.45 (6.03–44.84)
≥65		90.49 (32.88–249.09)	29.80 (10.56–84.15)	28.31 (10.01–80.04)	28.31 (10.01–80.05)
**Urbanization**					
Urban		Reference	Reference	Reference	Reference
Suburban		1.05 (0.79–1.40)	1.02 (0.76–1.36)	1.01 (0.76–1.35)	1.02 (0.77–1.37)
Rural		1.07 (0.76–1.50)	1.01 (0.72–1.41)	0.98 (0.70–1.38)	1.02 (0.73–1.43)
**Low income**		1.61 (1.21–2.15)	1.41 (1.05–1.90)	1.36 (1.01–1.82)	1.40 (1.05–1.88)
Length of hospital stays^*^					
0 day			Reference	Reference	Reference
1–6 days			1.66 (1.19–2.32)	1.66 (1.19–2.31)	1.66 (1.19–2.31)
≥7 days			1.84 (1.33–2.54)	1.81 (1.31–2.51)	1.79 (1.29–2.49)
**Co-morbidity^†^**					
Heart failure			1.50 (0.84–2.70)	1.41 (0.78–2.55)	1.48 (0.82–2.65)
Hypertension			1.83 (1.36–2.47)	1.77 (1.31–2.38)	1.85 (1.37–2.49)
Diabetes mellitus			1.70 (1.23–2.34)	1.73 (1.26–2.38)	1.70 (1.23–2.35)
Hyperlipidemia			1.42 (1.04–1.93)	1.44 (1.06–1.96)	1.43 (1.05–1.94)
Ischemic heart disease			1.97 (1.41–2.76)	2.02 (1.45–2.83)	2.01 (1.43–2.81)
Ischemic stroke			1.67 (0.99–2.83)	1.81 (1.07–3.07)	1.66 (0.98–2.80)
Pulmonary disease			1.20 (0.77–1.87)	1.13 (0.72–1.77)	1.21 (0.77–1.89)
Chronic kidney disease			1.65 (0.93–2.94)	1.68 (0.94–3.01)	1.62 (0.91–2.88)
Chronic liver disease			0.73 (0.39–1.38)	0.73 (0.39–1.39)	0.73 (0.39–1.39)
Hyperthyroidism			0.00 (0.00–6.02E286)	0.00 (0.00–2.02E177)	0.00 (0.00–1.75E287)
IBD			0.55 (0.07–4.25)	0.24 (0.03–1.97)	0.58 (0.08–4.52)
Psoriasis			0.42 (0.06–3.01)	0.36 (0.05–2.59)	0.43 (0.06–3.14)
**AS treatment during f/u period^‡^**					
NSAID, cDDD/day				1.35E166 (2.55E125–7.16E206)	
Methotrexate (cdose/2.5 mg) week				0.01 (0.00–1.25E36)	
Sulfasalazine (cdose/500 mg)/day				0.00 (0.00–0.00)	
Steroid, mg/day				17.20 (7.46–39.69)	
**AS treatment during f/u period^‡^**					
NSAID					0.73 (0.56–0.94)
Methotrexate					1.05 (0.45–2.46)
Sulfasalazine					0.79 (0.53–1.17)
Steroid					1.16 (0.87–1.56)

* *Length of hospital stay was identified within 2 years before index date*.

† *Comorbidity was identified within 1 year before index date*.

‡ *AS treatment was identified after diagnosis with AS*.

## Discussion

To the best of our knowledge, this is the largest cohort study using a longitudinal nationwide population-based dataset to identify the incidence risk of MACE for AS patients with AAU. The results of this study suggested that AS patients with AAU was not increase risk of MACE.

Previous studies have found that patients with AS may face an increased risk of cardiovascular disease (CVD) (5, 13). However, it is unclear whether all AS patients are at increased risk of CVD or are only related to specific risks. Berg et al. showed that AS patients with a history of uveitis have an increased risk of hypertension and atherosclerosis (14). Berg et al. observed that the sample size is small, and there may be deviations in the number of variances that may affect the results (14). Notwithstanding these previous observations, we studied the confirmation evidence of AS patients with AAU and the risk of MACE by using sensitivity analysis with more selective diagnostic methods, and these findings showed that the risk of MACE for AAU patients did not significantly different between two group. Although uveitis is a type of inflammation in AS patients, the pathophysiology of the relationship between uveitis and subsequent MACE is unclear. According to the prevalence of non-infected uveitis is 121 cases per 100,000 (95% CI = 117.5–124.3) and 29 per 100,000 for children (95% CI = 26.1–33.2) in the United States. Indeed, in Taiwan, the cumulative incidence of uveitis has risen, indicating 318.8 cases per 100,000 people in 2003 to 622.7 cases per 100,000 people in 2008. Moreover, the most common form of uveitis is anterior uveitis in Asia (15). In addition, our study is confirmed that AAU plays a neutral risk on the development of MACE in the population, confirming that the reliability is high and has clinical significance by longitudinal cohort designs.

Several reports have documented that TLR2 and TLR4 signaling pathways could induce vascular inflammation and insulin resistance, leading to a higher risk of atherosclerosis (16). On the contrary, it has been demonstrated that a significant reduction in TLR2 expression levels on monocytes and neutrophils of patients with AAU (14). In addition, interleukin-6 and IFN-gamma production stimulated by TLR4 activation was significantly reduced in patients with AAU (9). Previous reports have shown that there is associated between AS and cardiovascular disease. It may increase risk of cardiovascular comorbidity and mortality (17). Past studies have shown biomarkers of CVD in AS patients, the results showed that retinal vascular changes and impaired function (18, 19). We did not find a significant correlation between AS patients and the risk of MACE in AAU group. Our results also found that AS patients with hypertension, diabetes, hyperlipidemia were significantly associated with the development of MACE development. Furthermore, MTX use is associated with lower risk of cardiovascular events in patients with rheumatoid arthritis (RA) through the reduction of inflammation (18, 20). Sulfasalazine also plays an important role in protective anti-inflammatory effects against cardiovascular events in patients with autoimmune diseases, such as RA and AS (21). Meanwhile, several reports showed that NSAIDs use in clinical practice may decrease inflammation and have a negative effect on the cardiovascular disease in AS patients (22–24).

This study has several strengths. First, among AS patients, patients with AAU did not have higher risk of subsequent MACE by using a nationwide database to support this research. Second, 99% of the Taiwanese population (around 23 million residents) is enrolled in NHIRD, which has been validated by NHRI, selection bias of age, region and institution can be minimized. Our study analyzed a population-based sample of AS patients follow-up the long-term outcomes of this Taiwanese population. Third, we analyzed that AS patients with AAU was associated with neutral effect on MACE, which subgroup analysis was also conducted to improve clinical application, and we used age and sex matching and sensitivity tests to reduce confounding bias. Overall, we provided new insights into the association between AAU and MACE risk among AS patients. Our study has some limitations. Although we had adjusted for many covariates, some residual confounders might still exist. Thus, we compiled five models of sensitivity tests to confirm the robust findings. Furthermore, the NHIRD datasets lack some lifestyle data, such as smoking and alcohol consumption. Hence, we matched lifestyle-related comorbidities as proxies, including COPD, hypertension, and coronary heart disease to minimize this limitation. NSAID had been considered a risk of factor of MACE in the general population (25, 26). However, several reports showed that NSAIDs use in clinical practice might decrease inflammation and have a negative effect on cardiovascular disease in AS patients (22–24). In the Cox regression analysis, we found that use of NSAIDs during the follow-up period was associated with a decreased risk of MACE. However, this finding may be explained by immortal time bias or confounding by indication. First, immortal time refers to the follow-up period during which the outcome could not have occurred (unexposed and immortal) (27). Time-dependent Cox regression analysis is a common method to mitigate immortal time bias in the exposed group. We found that the HR of MACE associated with NSAID use during the follow-up period increased from 0.32 to 0.73 when we used the time-dependent Cox regression model instead, suggesting the existence of immortal time bias. Second, as shown in Supplementary Table 1A, patients who ever received NSAID treatment during the follow-up period had significantly lower proportions of heart failure ischemic stroke, which were known risk factors of MACE. Therefore, bias related to confounding by indication might also exist. Given that almost all AS patients received NSAID treatment during the follow-up period, calculating the HR of MACE associated with time-averaged doses of NSAIDs during the follow-up period in a Cox regression model may be a better way to assess the influence of NSAID on MACE. We found that the average daily dose of NSAID (cDDD/day) was associated with an increased risk of MACE (HR, 3.44). Therefore, we cannot make a conclusion on the association of NSAIDs with the risk of MACE based on the results of the study. Finally, the definition of International Uveitis Research Group (IUSG), the subtypes of uveitis included anterior uveitis, middle uveitis, posterior uveitis, and panuveitis (28). All the types of inflammation of uveitis can be divided into infectious and non-infectious (29–32). Our study focuses on non-infectious uveitis and the diagnosis of AAU is acceptable and stable, depending on our selection criteria.

## Conclusion

This retrospective cohort study demonstrated that AS patients with AAU did not increase the risk of subsequent MACE. Further studies are warranted to explore the mechanism of this association. We provided new insights into the association between AAU and MACE risk among AS patients.

## Data Availability Statement

The original contributions presented in the study are included in the article/Supplementary Material, further inquiries can be directed to the corresponding author.

## Ethics Statement

The studies involving human participants were reviewed and approved by the Institutional Review Board Chung Shan Medical University Hospital CSMUH No: CS15134. Written informed consent for participation was not required for this study in accordance with the national legislation and the institutional requirements.

## Author Contributions

Y-CB and H-HC: study design and manuscript preparation. C-HL, P-YL, and K-LL: statistical analysis and critical comment. All authors contributed to the article and approved the submitted version.

## Conflict of Interest

The authors declare that the research was conducted in the absence of any commercial or financial relationships that could be construed as a potential conflict of interest.

## Publisher's Note

All claims expressed in this article are solely those of the authors and do not necessarily represent those of their affiliated organizations, or those of the publisher, the editors and the reviewers. Any product that may be evaluated in this article, or claim that may be made by its manufacturer, is not guaranteed or endorsed by the publisher.
