# Supplementary material for: The Risk of Major Adverse Cardiovascular Events in Ankylosing Spondylitis Patients With a History of Acute Anterior Uveitis: A Nationwide, Population Based Cohort Study
Source: Front Med (Lausanne). 2022 Jul 7;9:884800. doi: 10.3389/fmed.2022.884800 (PMC9300849; doi:10.3389/fmed.2022.884800)
Supplement: Supplementary file 1 [file Data_Sheet_1.PDF]

Supplementary Table1. Characteristics among NSAID group and non-NSAID group.

	Before PSM			1:1 PSM		
	Non-NSAID n=647	NSAID n=17,073	P value	Non-NSAID n=274	NSAID n=6,806	P value
**Follow-up**	1.7±1.5	4.0±1.8	<0.001	1.5±1.5	3.9±1.8	<0.001
**AAU**	150 (23.2)	3,394 (19.9)	0.039	150 (54.7)	3,390 (49.8)	0.109
**Sex**			<0.001			<0.001
Female	157 (24.3)	6,028 (35.3)		67 (24.5)	2,405 (35.3)	
Male	490 (75.7)	11,045 (64.7)		207 (75.5)	4,401 (64.7)	
**Age**	44.1±14.7	40.8±13.8	<0.001	44.5±15.1	40.7±13.8	<0.001
<30	121 (18.7)	3,939 (23.1)	<0.001	50 (18.2)	1,572 (23.1)	<0.001
30-45	219 (33.8)	6,611 (38.7)		90 (32.8)	2,640 (38.8)	
45-65	250 (38.6)	5,630 (33.0)		107 (39.1)	2,241 (32.9)	
≥65	57 (8.8)	893 (5.2)		27 (9.9)	353 (5.2)	
**Urbanization**			0.298			0.234
Urban	215 (33.2)	5,375 (31.5)		104 (38.0)	2,254 (33.1)	
Suburban	323 (49.9)	8,424 (49.3)		128 (46.7)	3,370 (49.5)	
Rural	109 (16.8)	3,274 (19.2)		42 (15.3)	1,182 (17.4)	
**Low income (<=Q2:21,900)**	371 (57.3)	9,716 (56.9)	0.827	147 (53.6)	3,713 (54.6)	0.768
**Length of hospital stays***	4.2±22.3	2.3±14.9	0.027	2.3±11.7	2.0±14.9	0.671
0 day	539 (83.3)	14,147 (82.9)	0.047	231 (84.3)	5,791 (85.1)	0.641
1–6 days	48 (7.4)	1,654 (9.7)		23 (8.4)	609 (8.9)	
≥7 days	60 (9.3)	1,272 (7.5)		20 (7.3)	406 (6.0)	
**Co-morbidity†**						
Heart failure	9 (1.4)	92 (0.5)	0.005	3 (1.1)	36 (0.5)	0.215
Hypertension	89 (13.8)	2,025 (11.9)	0.144	34 (12.4)	818 (12.0)	0.846
Diabetes mellitus	39 (6.0)	799 (4.7)	0.113	13 (4.7)	274 (4.0)	0.554
Hyperlipidemia	54 (8.3)	1,438 (8.4)	0.945	21 (7.7)	514 (7.6)	0.945
Ischemic heart disease	23 (3.6)	532 (3.1)	0.529	10 (3.6)	166 (2.4)	0.207
Ischemic stroke	12 (1.9)	134 (0.8)	0.003	5 (1.8)	47 (0.7)	0.031
Pulmonary disease	16 (2.5)	522 (3.1)	0.395	5 (1.8)	199 (2.9)	0.286
Chronic kidney disease	9 (1.4)	143 (0.8)	0.134	1 (0.4)	35 (0.5)	0.733
Chronic liver disease	23 (3.6)	466 (2.7)	0.208	5 (1.8)	141 (2.1)	0.778
Hyperthyroidism	3 (0.5)	96 (0.6)	0.741	1 (0.4)	30 (0.4)	0.852
IBD	1 (0.2)	36 (0.2)	0.758	0 (0.0)	6 (0.1)	0.623
Psoriasis	4 (0.6)	131 (0.8)	0.669	1 (0.4)	44 (0.6)	0.565
APS	0 (0.0)	2 (0.01)	0.783	0 (0.0)	0 (0.0)	NA
**AS treatment at baseline‡**						
NSAID, cDDD/day	0.0±0.0	0.2±0.3	<0.001	0.0±0.0	0.2±0.3	<0.001
Methotrexate (1) (cdose/2.5mg)week	0.01±0.2	0.1±0.7	<0.001	0.005±0.1	0.1±0.4	<0.001

Sulfasalazine (2) (cdose/500mg)/day	0.1±0.3	0.3±0.5	<0.001	0.1±0.3	0.3±0.6	<0.001
Steroid, mg/day	0.4±3.2	0.5±6.4	0.588	0.5±2.5	0.5±1.8	0.785
Etanercept,200mg/4week	0.003±0.05	0.01±0.1	0.112	0.01±0.1	0.00±0.1	0.952
Adalimumab,80mg/4week	0.001±0.03	0.01±0.1	<0.001	0.0±0.0	0.01±0.1	<0.001
Golimumab,50mg/4week	0.002±0.04	0.0005±0.01	0.484	0.003±0.1	0.001±0.01	0.424
Methotrexate (1)	8 (1.2)	1,271 (7.4)	<0.001	2 (0.7)	563 (8.3)	<0.001
Sulfasalazine (2)	50 (7.7)	7,851 (46)	<0.001	28 (10.2)	3,684 (54.1)	<0.001
Steroid	143 (22.1)	10,183 (59.6)	<0.001	70 (25.5)	4,377 (64.3)	<0.001
Etanercept	3 (0.5)	263 (1.5)	0.027	3 (1.1)	130 (1.9)	0.33
Adalimumab	1 (0.2)	308 (1.8)	0.002	0 (0.0)	165 (2.4)	0.009
Golimumab	1 (0.2)	46 (0.3)	0.577	1 (0.4)	27 (0.4)	0.935

Supplementary Table2a. Incidence of MACE in PSM study group

	Before PSM		1:1 PSM	
	Non-NSAID	NSAID	Non-NSAID	NSAID
n	647	17,073	274	6,806
Follow-up person years	1,111	67,677	412	26,660
MACE	22 (3.40)	247 (1.45)	8 (2.92)	85 (1.25)
Incidence rate* (95%CI)	1,979.90 (1,977.28-1,982.51)	364.97 (364.82-365.11)	1,943.90 (1,939.64-1,948.16)	318.83 (318.62-319.05)
Crude relative risk (95%CI)	Ref.	0.18 (0.12-0.29)	Ref.	0.16 (0.08-0.34)
Average Follow-up duration	1.7	4.0	1.5	3.9

*Incidence rate, per 100,000 person-years

Supplementary Table2b. Incidence of MACE in PSM study group

	Before PSM		1:1 PSM	
	Non-MTX	MTX	Non-MTX	MTX
n	16,441	1,279	6,515	565
Follow-up person years	63,334	5,454	24,679	2,393
MACE	255 (1.55)	14 (1.09)	84 (1.29)	9 (1.59)
Incidence rate* (95%CI)	402.62 (402.47-402.78)	256.69 (256.27-257.12)	340.37 (340.14-340.61)	376.17 (375.40-376.95)
Crude relative risk (95%CI)	Ref.	0.64 (0.37-1.09)	Ref.	1.11 (0.56-2.20)
Average Follow-up duration	3.9	4.3	3.8	4.2

*Incidence rate, per 100,000 person-years

Supplementary Table2c. Incidence of MACE in PSM study group

	Before PSM		1:1 PSM	
	Non-SSZ	SSZ	Non-SSZ	SSZ
n	9,819	7,901	3,368	3,712
Follow-up person years	35,462	33,326	11,735	15,336
MACE	205 (2.09)	64 (0.81)	61 (1.81)	32 (0.86)
Incidence rate* (95%CI)	578.08 (577.83-578.33)	192.04 (191.89-192.19)	519.81 (519.40-520.23)	208.66 (208.43-208.89)
Crude relative risk (95%CI)	Ref.	0.33 (0.25-0.44)	Ref.	0.40 (0.26-0.62)
Average Follow-up duration	3.6	4.2	3.5	4.1

*Incidence rate, per 100,000 person-years
